# Symptom Burden After Acute Pancreatitis and Its Correlation With Exocrine Pancreatic Function: A Multicenter Prospective Study

**DOI:** 10.14309/ctg.0000000000000799

**Published:** 2024-12-16

**Authors:** Joseph Bejjani, Stacey Culp, Melica Nikahd, Anna Evans Phillips, Vikesh Singh, Kristen M. Roberts, Maisam Abu-El-Haija, Somashekar G. Krishna, Mitchell L. Ramsey, Ali Lahooti, Peter J. Lee, Phil A. Hart, Georgios I. Papachristou

**Affiliations:** 1 Division of Gastroenterology, Hepatology, and Nutrition, Wexner Medical Center, The Ohio State University, Columbus, Ohio, USA;; 2 Department of Biomedical Informatics, Center for Biostatistics, The Ohio State University Wexner Medical Center, Columbus, Ohio, USA;; 3 Department of Medicine, Division of Gastroenterology, Hepatology, and Nutrition, University of Pittsburgh School of Medicine, Pittsburgh, Pennsylvania, USA;; 4 Department of Medicine, Division of Gastroenterology and Hepatology, Johns Hopkins Medical Institutions, Baltimore, Maryland, USA;; 5 School of Health and Rehabilitation Sciences, The Ohio State University, Columbus, Ohio, USA;; 6 Department of Pediatrics, College of Medicine, University of Cincinnati, Cincinnati, Ohio, USA; and; 7 Division of Pediatric Gastroenterology, Hepatology and Nutrition, Cincinnati Children's Hospital Medical Center, Cincinnati, Ohio, USA.

**Keywords:** acute pancreatitis, exocrine pancreatic insufficiency, gastrointestinal symptoms, weight loss

## Abstract

**INTRODUCTION:**

Gastrointestinal (GI) symptoms and weight loss develop during and after acute pancreatitis (AP), but remain understudied. In this prospective, multicenter study, we aim to assess GI symptom burden and weight loss and their correlation with exocrine function up to 12 months post-AP.

**METHODS:**

GI symptom burden, anthropometrics, and exocrine pancreatic function were systematically measured in adults (≥18 years) with AP at predefined intervals: hospitalization (enrollment), 3 months, and 12 months post-AP. Symptoms were evaluated using a 15-item tracker, including abdominal symptoms, stool characteristics, and activities of daily living, higher scores indicating greater symptom burden (range 0–45). Exocrine function was assessed with fecal elastase-1 (FE-1) levels.

**RESULTS:**

GI symptoms were collected in 97 participants with 12-month follow-up. The median (interquartile range) GI-symptom score was 7 (3–12) with 55 participants (57%) experiencing at least one symptom frequently (often or almost always). In multivariable linear regression, younger age, lower Charlson Comorbidity Index, smoking, recurrent AP, and alcoholic or idiopathic etiologies were associated with significantly higher GI-symptom burden at 12 months. A significant negative correlation was found between GI symptoms and FE-1 levels during hospitalization (*ρ* = −0.288; *P* = 0.015) and at 12 months (*ρ* = −0.219; *P* = 0.046). Eighteen participants (18.6%) lost ≥10% body weight between hospitalization and 12 months, and had significantly lower median FE-1 levels at 12 months compared with the group without weight loss (166 vs 332 µg/g, *P* = 0.016).

**DISCUSSION:**

This is the first study to prospectively assess GI-symptom burden and exocrine function post-AP. Lower exocrine pancreatic function at 12 months was associated with increased symptom burden and weight loss. These findings support further investigations to define and improve patient-reported outcomes post-AP. This study is registered with ClinicalTrials.gov, NCT03063398.

## Graphical abstract

**Figure:**
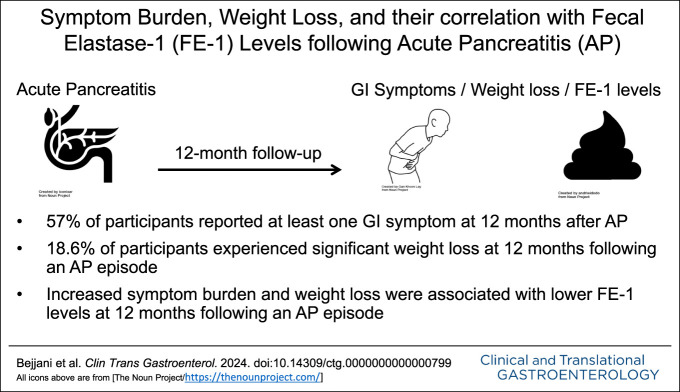

## INTRODUCTION

Acute pancreatitis (AP) is one of the leading gastrointestinal (GI) causes for hospital admissions resulting in health care costs that reach $3 billion annually in the United States alone (1). Traditionally, it has been assumed that recovery from AP leads to the full restoration of pancreatic function, except in situations where extensive pancreatic necrosis has occurred (2). Consequently, patients with uncomplicated AP, meaning without any local or systemic complications, have not been consistently followed up after discharge. However, there is a growing recognition of long-term consequences after an AP episode in the exocrine and endocrine pancreatic function, suggesting the need for systemic follow-up to further study the posthospital course (3–5).

Recent studies have revealed that exocrine pancreatic insufficiency (EPI) occurs in a significant percentage of patients after an AP episode (6,7). Meta-analyses conducted by Huang et al and Hollemans et al (6,7) have indicated that the point prevalence of EPI is 62% during hospitalization. In addition, persistent EPI has been observed in 25%–35% of patients during long-term follow-up, with recovery occurring at varying intervals (2). The incidence of EPI after AP is believed to be higher in cases with alcoholic etiology, organ failure, and pancreatic necrosis (6). However, EPI can still develop in patients with mild AP, with rates of 20% during follow-up (7).

Currently, there are no societal guidelines on assessment of EPI after AP. Historically, this has been challenging, partly due to a lack of consensus on definitive diagnostic criteria (8). Exocrine pancreatic dysfunction (EPD) is a term used to describe a reduction of the exocrine function of the pancreas in the absence of formal EPI diagnosis. Weight loss, abdominal pain, diarrhea, and other GI symptoms can develop after AP, representing the most prominent clinical findings of EPD. In a critical review of the literature, Durie et al proposed the use of weight loss and GI symptoms as indicators of EPD (9). Furthermore, the association of GI symptoms and weight loss with fecal elastase-1 (FE-1) levels has not been previously investigated. Recently, Phillips et al (10) reported that weight loss is seen in one-quarter of patients and therefore concluded that EPD incidence after AP is likely underestimated.

There is a lack of prospective data on the burden of GI symptoms after an AP attack. The aim of this study was to evaluate the burden of GI symptoms, identify the risk factors associated with the persistence of these symptoms, and examine the correlation between GI symptoms, weight loss, and fecal elastase-1 levels at 12 months post-AP in a prospective cohort study.

## METHODS

### Study design and eligibility criteria

The Post-Acute Pancreatitis Pancreatic Exocrine Insufficiency (PAPPEI) Study is a prospective, observational study conducted at The Ohio State University Wexner Medical Center, the University of Pittsburgh Medical Center, and Johns Hopkins University Medical Center (NCT03063398). The study was approved by the local Institutional Review Boards and conducted from June 2017 to October 2022.

The protocol of the PAPPEI study has been previously described (11). In brief, adults(≥18 years) hospitalized with AP were enrolled, then completed 3 visits at baseline (during hospitalization for AP), 3 months, and 12 months after their AP diagnosis with serial data and biosample collections at each. AP diagnosis was defined according to the revised Atlanta classification (12). Eligibility was determined by reviewing medical records and conducting interviews. Exclusion criteria included a previous diagnosis of EPI, chronic pancreatitis, pancreatic cancer; prior pancreatic resection; and history of gastric bypass, gastroparesis, cystic fibrosis, and malabsorptive disorder (including inflammatory bowel disease and celiac disease). Informed consent was provided before any study procedures. Data regarding participant and disease-related characteristics were gathered using standardized case report forms. Information was collected from the Electronic Medical Record and by interviewing the study participants. Active alcohol use or smoking and former alcohol use or smoking were defined as within the past 6 months and more than 6 months ago, respectively. Never smoking was defined as <100 cigarettes or 5 packs in a lifetime, while those who had <20 drinks in a lifetime were classified as never for alcohol use.

### GI-symptom testing

GI symptoms were evaluated using a 15-item symptom questionnaire, including abdominal symptoms, stool characteristics, and activities of daily living (see Supplementary Table 1, http://links.lww.com/CTG/B235). The GI symptom tracker was developed through qualitative focus group interviews of participants with EPI (10). Responses are scored as almost always = 3, “often” = 2, sometimes = 1, or never = 0. The total symptom score is the sum of all responses (range: 0–45; higher scores indicating a greater symptom burden). For part of the following analysis, we dichotomized the responses into frequent (almost always/often) and not frequent (sometimes/never) and grouped patients by whether or not they experienced at least one frequent symptom at 12 months after AP.

### Weight assessment

Body weight was recorded from the Electronic Medical Record at baseline. During the 3-month and 12-month follow-up periods, participants were asked if they had experienced unintentional weight loss, and the amount of weight loss was documented. Involuntary weight loss of ≥10% of baseline weight is generally considered to represent protein-energy malnutrition and therefore was determined as clinically relevant in our analyses. This determination has been supported by the association between this degree of weight loss (10%), nutritional deficiencies, and poor wound healing in general (13).

### Fecal elastase testing

Stool samples were collected at baseline, 3 months, and 12 months, and then analyzed for FE-1 concentration using enzyme-linked immunosorbent assay quantifying CELA2/CELA3 isoforms (JOLI Diagnostic Inc., Williamsville, NY). In our study, participants were instructed to send formed stool samples. However, FE-1 levels were measured regardless of stool consistency. For the purposes of this study, FE-1 > 200 µg/g stool was considered normal, and EPI was operationalized as FE-1 ≤ 200 µg/g stool for analysis; values 100 < FE-1 level ≤ 200 µg/g stool were considered mild EPI, and FE-1 level ≤ 100 µg/g stool was considered severe EPI.

### Statistical analysis

Baseline characteristics were summarized using means and standard deviations for continuous variables and relative frequencies for categorical variables. Data were divided into 2 groups for initial analysis, creating a categorical GI symptom group variable with 2 levels: participants with one or more GI symptoms occurring frequently at the 12 months assessment and participants with no GI symptoms occurring frequently at 12 months after AP. Independent samples *t*-tests were used to compare means of continuous variables by GI symptom group, and χ^2^ tests or the Fisher exact tests were used to assess the association between GI symptom group and categorical variables. Demographics and disease characteristics at baseline with a statistically significant difference (*P* < 0.05) by GI symptom group at 12 months were used in a multivariable linear regression analysis to identify independent risk factors associated with having at least one GI symptom frequently at 12 months. In addition, the percentage of patients experiencing a symptom frequently at 12 months was calculated for each individual symptom, and Cochran *Q* test was used to compare the proportion of participants frequently experiencing at least one symptom across time points.

The results from the GI symptom tracker were also used as a continuous variable, henceforth GI symptom score. The correlations between GI symptom scores and FE-1 levels at baseline and 12 months were calculated using the Spearman *ρ* correlation coefficient. A nonparametric test was adopted because FE-1 data were truncated at 50 µg/g on the lower end and 500 µg/g on the upper end. The Spearman *ρ* was also used to assess the correlation between the ordinal FE-1 variable (FE-1 ≤ 100 µg/g; 100 <FE-1 ≤ 200 µg/g; FE-1 >200 µg/g) and GI symptom scores. In addition, the Freidman test was used to compare GI symptom scores over time for participants with data at all 3 time points.

Percent weight loss between baseline and 12 months follow-up was calculated for each participant and was dichotomized into 2 groups: those who experienced weight loss ≥10% and those who did not. Mean GI symptom scores were compared between the 2 weight loss groups using an independent samples *t*-test, while a Mann-Whitney *U* test was used to compare FE-1 levels by weight loss group. A 1-sided χ^2^ test was used to assess the relationship between the ordinal FE-1 variable and weight loss group. All statistical analyses were completed in IBM SPSS Statistics version 28.0 (Armonk, NY). A *P* value of <0.05 was considered statistically significant.

## RESULTS

### Baseline characteristics

A total of 97 participants completed the GI symptom tracker questionnaire at 12 months after enrollment and were selected for analysis of the primary end point. Most participants in the study population were White (90%), 52% were female, and 54% were obese (body mass index ≥ 30 kg/m^2^). Gallstone was the most common etiology of AP, seen in 38% of participants. This was the first episode of AP for most participants (62%), and the majority (63%) experienced mild clinical severity (Table 1).

**Table 1.** Baseline demographics and disease characteristics by the presence of GI symptoms at 12 mo from acute pancreatitis

Variable	Level	Total participants (n = 97)	Persistent GI symptoms (n = 55)	No GI symptoms (n = 42)	*P* value
		Mean (SD)	Mean (SD)	Mean (SD)	
Age (yr)		52.6 (14.3)	48.9 (14.5)	57.4 (12.7)	0.003
Charlson Comorbidity Index		2.8 (1.7)	2.5 (1.4)	3.3 (1.9)	0.021
		n (%)	n (%)	n (%)	
Sex	Male	47 (48.5)	26 (47.3)	21 (50.0	0.790
	Female	50 (51.5)	29 (52.7)	21 (50.0)	
Race	White	87 (89.7)	48 (87.3)	39 (97.2)	0.507*
	Black	6 (6.2)	5 (9.1)	1 (2.4)	
	Multiple, other, declined to respond	4 (4.1)	2 (3.6)	2 (4.8)	
BMI	Nonobese	45 (46.4)	28 (50.9)	17 (40.5)	0.307
	Obese (≥30 kg/m^2^)	52 (53.6)	27 (49.1)	25 (59.5)	
Preexisting diabetes	Yes	30 (30.9)	17 (30.9)	13 (31.0)	1.000
	No	67 (69.1)	38 (69.1)	29 (69.0)	
Smoking	Never	50 (52.1)	27 (49.1)	23 (56.1)	0.023
	Active	16 (16.7)	14 (25.5)	2 (4.9)	
	Former	30 (31.3)	14 (25.5)	16 (39.0)	
Alcohol use	Never	24 (24.7)	14 (25.5)	10 (23.8)	0.561
	Active	46 (47.4)	28 (50.9)	18 (42.9)	
	Former	27 (27.8)	13 (23.6)	14 (33.3)	
History of AP	No (first episode)	60 (61.9)	27 (49.1)	33 (78.6)	0.003
	Yes (recurrent episode)	37 (38.1)	28 (50.9)	9 (21.4)	
AP etiology	Gallstones	37 (38.1)	15 (27.3)	22 (52.4)	0.020**
	Alcoholic	11 (11.3)	10 (18.2)	1 (2.4)	
	Idiopathic	28 (28.9)	18 (32.7)	10 (23.8)	
	Hypertriglyceridemia	8 (8.2)	5 (9.1)	3 (7.1)	
	Post-ERCP	6 (6.2)	3 (5.5)	3 (7.1)	
	Other	7 (7.2)	4 (7.3)	3 (7.1)	
Severity of AP	Mild	61 (62.9)	31 (56.4)	30 (71.4)	0.314
	Moderately severe	27 (27.8)	18 (32.7)	9 (21.4)	
	Severe	9 (9.3)	6 (10.9)	3 (7.1)	
(Peri)Pancreatic necrosis (n = 71)	Yes	17 (23.9)	11 (26.8)	6 (20.0)	0.505
	No	54 (76.1)	30 (73.2)	24 (80.0)	

AP, acute pancreatitis; BMI, body mass index; EPI, exocrine pancreatic insufficiency; ERCP, endoscopic retrograde cholangiopancreatography; GI, gastrointestinal.

* *P* value corresponds to the Fisher exact test for White vs not White due to cell counts.

** *P* value corresponds to a χ^2^ test using a 4-level etiology variable (gallstones, alcoholic, idiopathic, other) due to cell counts.

### Predictors of frequent GI symptoms at 12 months

Baseline demographics and disease characteristics were compared between participants who frequently experienced at least one GI symptom at 12 months (GI symptom group: n = 55) and those who did not (n = 42). Participants with persistent GI symptom(s) were more likely to be younger, with fewer comorbidities (lower Charlson Comorbidity Index, be active smokers, and have a history of AP (Table 1). In addition, regarding the distribution of AP etiologies, a higher percentage of idiopathic or alcoholic etiology was observed in participants with GI symptom(s). Although a higher proportion of participants with GI symptom(s) at 12 months had moderately severe or severe AP (43.6% vs 28.5%), this difference did not reach statistical significance.

Based on the univariable results, age, Charlson Comorbidity Index, and history of AP were included in the multivariable linear regression model to identify the contribution of each factor to the GI symptom score at 12 months. In addition, alcohol and idiopathic etiologies were included in the model as a single entity. Current and past smoking status were also grouped together and included in the linear regression analysis. In the multivariable model, age, smoking status, AP history, and etiology were significant predictors of GI symptom score at 12 months with higher scores being observed in younger participants, active or former smokers, those with a previous history of AP, and those with alcohol or idiopathic etiology (Table 2).

**Table 2.** Multivariable linear regression of risk factors associated with GI symptoms at 12 mo after AP episode

Variable	β	SE (β)	*t* statistic	*P* value
Intercept	15.92	2.92	5.44	<0.001
Age in yr	−0.22	0.07	−3.25	0.002
Charlson comorbidity index	0.06	0.57	0.11	0.913
History of AP	3.40	1.53	2.22	0.029
Alcohol or idiopathic etiology	3.01	1.51	2.00	0.049
Smoke ever	3.46	1.35	2.56	0.012

Model fit: *R*^2^ = 0.33.

AP, acute pancreatitis; GI, gastrointestinal.

Of the 55 participants with frequent GI symptom(s) at 12 months, 31% (n = 17) experienced 1 symptom, 22% (n = 12) experienced 2 symptoms, 7% (n = 4) experienced 3 symptoms, 15% (n = 8) experienced 4 symptoms (15%), and 25% (n = 14) experienced 5 or more symptoms frequently. Loose stools were the most commonly occurring symptom, reported by 49% (n = 27) of participants with GI symptom(s) at 12 months. This was followed by symptoms of feeling bothered by eating fatty food in 22 (40%) and bloating in 18 (33%) (Table 3).

**Table 3.** Prevalence of gastrointestinal symptoms at 12 mo among 55 participants with at least one symptom occurring on a frequent basis (often or almost always)

Symptom occurring frequently at 12 mo	Participants, n (%)
Loose stools	27 (49.1)
Bothered/concerned by eating fatty or greasy foods	22 (40.0)
Bloated	18 (32.7)
Excessive gas	16 (29.1)
Abdominal pain	16 (29.1)
Bothered/concerned by having a poor appetite because of GI problems	16 (29.1)
Frequent diarrhea	12 (21.8)
Foul smelling stool	12 (21.8)
Bothered/concerned by using a public bathroom	11 (20.0)
Bothered/concerned by missed daily activities due to GI problems	11 (20.0)
Bothered/concerned by skipping a meal	10 (18.2)
Bothered/concerned by staying on the toilet for a long time	7 (12.7)
Greasy/oily stools	6 (10.9)
Rush to the bathroom in the night	6 (10.9)
Difficult to flush stool	4 (4.3)

### Natural history of GI symptoms over 12 months

The temporal evolution of GI symptoms was examined in an analysis of participants who completed the GI symptom tracker questionnaire at all 3 time points (n = 74). The median (interquartile range) GI symptom score decreased from 10 (5–15) at baseline, to 6 (2–12) at 3 months, and to 7 (3–12) at 12 months (*P* = 0.01). The prevalence of 1 or more GI symptom occurring frequently also decreased from 74% at baseline, to 58% at 3 months, and to 55% at 12 months (*P* = 0.011). Of the 55 participants with at least one frequent GI symptom at baseline, approximately 42% (n = 23) recovered over the subsequent 12 months: 18 recovered by 3 months, but 5 redeveloped at least one frequent GI symptom by 12 months with an additional 10 recovered by 12 months. New occurrence of at least one frequent GI symptom was seen in 6 participants between baseline and 3 months, and 4 additional participants between 3 and 12 months.

### Correlations between GI symptom scores and FE-1 levels

FE-1 levels were measured at baseline for 77 participants and at 12 months for 83 participants with the median (interquartile range) values of 249 (79–431) µg/g and 300 (128–417) µg/g, respectively (Table 4). There was a significant, negative correlation between FE-1 levels and GI symptom scores at baseline (*ρ* = −0.288; *P* = 0.015). We also observed a significant, negative correlation between FE-1 levels and GI symptom scores at 12 months (*ρ* = −0.219; *P* = 0.046).

**Table 4.** Gastrointestinal symptoms, weight loss, and FE-1 levels at baseline, 3, and 12 mo after an episode of acute pancreatitis

	Baseline	3 mo	12 mo
Participants with frequently occurring GI symptoms, n (%)^a^	67 (75.3)	45 (56.3)	55 (56.7)
Median GI symptom score (IQR)	10 (5–15)	6 (2–12)	7 (3–12)
Mean weight (SD), kg	96.1 (37.5)	93.6 (34.5)	92.7 (30.9)
Participants with 10% weight loss, n (%)	NA	14 (17.5)	18 (18.6)
Median FE-1 level (IQR), µg/g^b^	249 (79–431)	304 (117–411)	300 (128–417)

AP, acute pancreatitis; EPD, exocrine pancreatic dysfunction; EPI, exocrine pancreatic insufficiency; FE-1, fecal elastase-1; GI, gastrointestinal; IQR, interquartile range; NA, not applicable.

a GI symptoms and weight were derived from 89 questionnaires completed at baseline, 80 at 3 mo, and 97 at 12 mo.

b Median FE-1 level was based on 77 samples collected at baseline, 75 at 3 mo, and 83 at 12 mo.

### Body weight and FE-1 levels

The mean (SD) body weight of the study population was 96.1 (37.5) kg at baseline and decreased to 92.7 (30.9) kg at 12 months (Table 4). A total of 18.6% (18/97) of participants had significant weight loss (≥ 10%) at 12 months. These participants significant weight loss (n = 13) had significantly lower FE-1 levels at 12 months compared with participants who did not (n = 70; 166 µg/g vs 332 µg/g, respectively; *P* = 0.016). Of the participants who experienced significant weight loss at 12 months, 54% (7/13) had FE-1 levels ≤200 µg/g compared with 29% of those who did not experience weight loss (*P* = 0.037). However, there was no significant difference in GI symptom scores at 12 months between participants who did vs did not lose weight (*P* = 0.266).

## DISCUSSION

This is the first prospective study that systematically assessed the natural history and burden of GI symptoms after an episode of AP. We applied a 15-item GI-symptom questionnaire that includes abdominal symptoms, stool characteristics, and disruption of daily activities due to symptoms. We found that over half of the participants frequently experienced one or more of these symptoms at 12 months from their AP episode. Having loose stools and being bothered by eating fatty foods were the most commonly reported symptoms. Independent risks for having persistent GI symptoms included younger age, smoking, history of recurrent AP attacks, and idiopathic or alcoholic AP etiology. Importantly, GI symptoms were negatively correlated with FE-1 levels at 12 months, suggesting that impaired exocrine function may be a key contributor to symptoms.

In addition to symptom patterns, we observed that nearly 20% of participants experienced significant (>10%) body weight loss at 12 months from their AP episode. This finding is consistent with a previous study by Phillips et al (10), where 24% of subjects experienced significant weight loss at 12 months after AP. We observed a significant association between FE-1 levels and weight loss, suggesting that impaired exocrine pancreatic function may contribute to weight loss after AP. Other potential contributors to weight loss, particularly immediately after discharge, include suboptimal dietary intake due to persistent symptoms and/or diuresis (14). We did not find a correlation between weight loss and GI symptom burden, which may be partly explained by the comprehensive nature of the symptom questionnaire, which included concepts not directly related to exocrine function. In addition, AP severity and the presence of pancreatic necrosis were not found to be predictors of increased GI symptoms at 12 months. This lack of association could be explained by the small numbers of patients with pancreatic necrosis or severe disease in our cohort (n = 17 and 9, respectively) and by the extensive nature of the questionnaire that included concepts not solely related to pancreatic pathology. These observations indicate that there is an ongoing need for continued work to understand other contributing factors to signs and symptoms aside from EPD.

GI symptoms such as abdominal pain, bloating, loose stools, and weight loss represent common clinical manifestations of EPD, reflecting scenarios where exocrine function testing should be considered. However, it is also important to mention that other contributors to GI symptoms maybe present after AP, such as chronic inflammation with cytokine release (15), endocrine pancreatic dysfunction (16), and small intestinal bacterial overgrowth (17). Other experts have recently recommended systematic screening for EPD after AP irrespective of symptoms (18). They recommended FE-1 as the test of choice for EPI assessment to be performed initially after hospital discharge and then annually. At the same time, it is important to note that diagnostic testing for EPD is complex and there is no currently available accurate and convenient approach to testing (19). In the setting of these limitations in the field, we elected to use FE-1 levels in this study to simulate current practices aiming for our results to be applicable to physicians managing patients with AP. We acknowledge that this test is relatively inaccurate in diagnosing mild EPI (20,21). In addition, it is important for clinicians to be aware of other potential contributors to symptoms in this patient population, which can include, but are not limited to delayed complications of AP (e.g., a symptomatic peripancreatic fluid collection), gut dysmotility, medication side effects, postcholecystectomy changes, and antibiotic associated diarrhea. Our study provides prospective, high-quality evidence on the persistence of GI symptoms and body weight changes related to EPD. Additional evidence needs to be accumulated on surveillance programs and subsequently on interventions in long-term outcomes after AP. These data also provide a useful benchmark for future studies and trials investigating patient-reported outcomes in this patient population after hospitalization.

The findings of our prospective study support the notion that the incidence of EPD after AP is underappreciated. Our study identified subgroups of patients who are at higher risk of experiencing GI symptoms based on their baseline characteristics at the time of the AP episode. Specifically, younger age, smoking, recurrent AP attacks, alcoholic, and idiopathic etiologies were independently associated with the presence of GI symptoms at 12 months. Thus, characteristics and lifestyle habits unrelated to pancreatic diseases appear to partially contribute to the GI-symptom burden. The above findings suggest that certain subsets of patients, based on their risk factors and type of GI symptoms, may benefit from individualized diet counseling or therapies such as pancreatic enzyme replacement therapy or pain modulators after an AP attack.

Our multicenter study provides prospective, high-quality evidence on the persistence of GI symptoms and body weight changes and relationship to EPD up to one year after AP. However, there are also some limitations to consider. First, participants were recruited at 3 tertiary care centers, so there may be selection bias toward AP patients with more complicated clinical course. Second, participants were informed at enrollment of the need for 2 follow-up visits; therefore, a lower participation rate was seen in individuals who anticipated challenges with their follow-up, also contributing to potential selection bias. There was a considerable drop-out rate of 34% during the 12-month follow-up duration between participants who filled the questionnaires and provided stool samples on enrollment compared with those who completed the 12-month follow-up. This could be explained, in part, by the fact that the PAPPEI study was conducted during the COVID-19 pandemic, and as a result, a large amount of enrollment and follow-up visit schedules were disrupted (many encounters happened virtually and led to limited stool sample collections). In addition, in our study, FE-1 levels were measured regardless of stool samples consistency, which may have introduced false-positive FE-1 results. Although the symptom questionnaire used in this study has not undergone psychometric evaluation to assess its validity and reliability, it was developed by content experts and has been used in recent studies (10), so the results remain useful for comparison. Last, the body weight data were self-reported during follow-up visits, which may introduce recall and social desirability bias.

To our knowledge, this is the first study to prospectively evaluate the burden of GI symptoms and their correlation with weight loss and exocrine pancreatic function after AP. The persistence of GI symptoms at 12 months was common and associated with younger age, smoking history, previous episodes of AP, and alcohol or idiopathic etiology. Importantly, a significant negative association was found between FE-1 levels and GI symptoms during hospitalization and at 12 months post-AP, suggesting that exocrine dysfunction is a primary contributor to symptoms. Additional research is needed to refine the clinical approach to screening for and managing EPD and other causes of symptoms after AP.

## CONFLICTS OF INTEREST

**Guarantor of the article:** Georgios I. Papachristou, MD, PhD, FACG.

**Specific author contributions:** Study Concept and Design: J.B., S.C., M.N., A.E.P., V.S., A.L., P.A.H., G.I.P. Acquisition of Data, Drafting of Manuscript, Critical Revision of the Manuscript for Important Intellectual Content, Approval of Final Manuscript: J.B., S.C., M.N., A.E.P., V.S., K.M.R., M.A.-E.-H., S.G.K., M.L.R., A.L., P.J.L., P.A.H., G.I.P.

**Financial support:** The study was supported by an investigator-initiated grant (GIP) provided by AbbVie, Inc. Research reported in this publication was also supported by the National Institute of Diabetes and Digestive and Kidney Diseases (NIDDK) under award number U01DK127388 (to The Ohio State University), U01DK127377 (to University of Pittsburgh), U01DK127400 (to Johns Hopkins University). The content is solely the responsibility of the authors and does not necessarily represent the official views of the National Institutes of Health.

**Potential competing interests:** Georgios Papachristou has received research support from AbbVie, Inc. The remaining authors do not have potential conflicts of interest to disclose.

**Study Highlights**

**WHAT IS KNOWN**

✓ Gastrointestinal symptoms and weight loss are common during or after acute pancreatitis.
✓ The characterization of gastrointestinal symptoms after acute pancreatitis and their correlation with exocrine pancreatic function remains poorly studied.

**WHAT IS NEW HERE**

✓ Fifty-seven percent of participants experienced at least one gastrointestinal symptom frequently at 12 months after acute pancreatitis.
✓ Increased symptom burden and weight loss were both associated with impaired exocrine pancreatic function, manifested as lower fecal elastase-1 levels, at 12 months after acute pancreatitis.

## Supplementary Material
