# Supplementary material for: Symptom Burden After Acute Pancreatitis and Its Correlation With Exocrine Pancreatic Function: A Multicenter Prospective Study
Source: Clin Transl Gastroenterol. 2024 Dec 16;16(2):e00799. doi: 10.14309/ctg.0000000000000799 (PMC11845210; doi:10.14309/ctg.0000000000000799)
Supplement: Supplementary file 1 [file ct9-16-e00799-s001.docx]

|  | Almost Always | Often | Sometimes | Never |
| --- | --- | --- | --- | --- |
| 1. Loose stools |  |  |  |  |
| 1. Bothered/concerned by eating fatty or greasy foods |  |  |  |  |
| 1. Bloated |  |  |  |  |
| 1. Excessive gas |  |  |  |  |
| 1. Abdominal pain |  |  |  |  |
| 1. Bothered/concerned by having a poor appetite because of GI problems |  |  |  |  |
| 1. Frequent diarrhea |  |  |  |  |
| 1. Foul smelling stool |  |  |  |  |
| 1. Bothered/concerned by using a public bathroom |  |  |  |  |
| 1. Bothered/concerned by missed daily activities due to GI problems |  |  |  |  |
| 1. Bothered/concerned by skipping a meal |  |  |  |  |
| 1. Bothered/concerned by staying on the toilet for a long time |  |  |  |  |
| 1. Greasy/oily stools |  |  |  |  |
| 1. Rush to the bathroom in the night |  |  |  |  |
| 1. Difficult to flush stool |  |  |  |  |

**Supplementary Table 1.** Gastrointestinal symptoms questionnaire
